# Robust Multi-Dimensional Time Series Forecasting

**DOI:** 10.3390/e26010092

**Published:** 2024-01-22

**Authors:** Chen Shen, Yong He, Jin Qin

**Affiliations:** State Key Laboratory of Public Big Data, College of Computer Science and Technology, Guizhou University, Guiyang 550025, China; gs.dshen21@gzu.edu.cn (C.S.); qin_gs@163.com (J.Q.)

**Keywords:** multidimensional time series forecasting, *L*_2,1_ norm, nonnegative matrix factorization (NMF), robust

## Abstract

Large-scale and high-dimensional time series data are widely generated in modern applications such as intelligent transportation and environmental monitoring. However, such data contains much noise, outliers, and missing values due to interference during measurement or transmission. Directly forecasting such types of data (i.e., anomalous data) can be extremely challenging. The traditional method to deal with anomalies is to cut out the time series with anomalous value entries or replace the data. Both methods may lose important knowledge from the original data. In this paper, we propose a multidimensional time series forecasting framework that can better handle anomalous values: the robust temporal nonnegative matrix factorization forecasting model (RTNMFFM) for multi-dimensional time series. RTNMFFM integrates the autoregressive regularizer into nonnegative matrix factorization (NMF) with the application of the $L_{2,1}$ norm in NMF. This approach improves robustness and alleviates overfitting compared to standard methods. In addition, to improve the accuracy of model forecasts on severely missing data, we propose a periodic smoothing penalty that keeps the sparse time slices as close as possible to the time slice with high confidence. Finally, we train the model using the alternating gradient descent algorithm. Numerous experiments demonstrate that RTNMFFM provides better robustness and better prediction accuracy.

## 1. Introduction

With the advancement of Internet of Things (IoT) technology and the reduced cost of sensor deployment, numerous IoT applications are producing massive amounts of time series data, such as intelligent building energy monitoring [1] and real-time rail monitoring applications [2]. Users can get information about the monitored object or region in real time according to multiple sensors, enhancing the effectiveness and security of pertinent decisions. Large size and multidimensionality are two important characteristics of the time series data used in these applications. For example, a smart building might include hundreds of sensors that track energy consumption [1]. Typically, such time series are sampled at high frequencies over lengthy periods of time, which can be leveraged to forecast energy consumption patterns, detect anomalies, and optimize building management decisions. Another feature is that most of these data have non-negativity constraints. For example, the speed of vehicle movement and customer electricity consumption are usually stored in a nonnegative matrix. In addition to IoT data, multidimensional time series data are similarly generated in some fields, such as e-commerce [3], web traffic [4], and the biomedical field [5].

**Figure 1:**
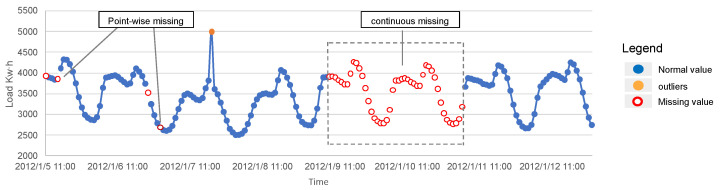
Illustration of two data missing patterns and outliers.

The development of modern sensor technology and large-scale data storage technology has brought some new challenges to the task of multidimensional time series forecasting. First, while the data may be generated by different sensors or objects, it may be impacted by common trends [6]. Traditional statistical time series forecasting models like ARIMA [7] are hard to build correlations across dimensions. Second, sensor data are often plagued by data loss problems, power exhaustion of sensor nodes, monitoring objects temporarily disappearing, and network congestion are all important causes of data loss. In the actual world, we typically observe two types of missing data patterns: pointwise missing, where data is lost randomly, and continuous missing, in which data is lost continuously for a period of time. Further, noise and outliers are prevalent in the collection and transmission of data due to factors such as electromagnetic interference or various external, immeasurable disturbances [8]. Severe outliers can change the distribution of the original data, so pre-processing for outliers and missing values is required before performing the prediction task. Yet, the accuracy of the preprocessed data directly affects the performance of the prediction model, and the high-quality interpolation algorithm incurs additional computational costs. Figure 1 illustrates two data missing patterns and outliers, where missing entries are marked with hollow dots. Outlier entries are marked with yellow dots.

To address these challenges, we focus on several recent approaches based on matrix factorization (MF) to solve these problems in multidimensional time series data forecasting. Matrix factorization can find a lower-dimensional representation of the original matrix that captures the latent features or patterns in the data. To enable matrix factorization models to be applied in time series forecasting applications [9], Yu et al. [3] provided temporal regularization matrix factorization (TRMF) that accounts for positive and negative correlations by placing a new AR regularizer on the temporal factor matrix. Ahn et al. [10] used a Gaussian kernel as a weighting function to model temporal factors with temporal correlation. Overall, these matrix factorization methods can automatically solve the missing value problem, and they have demonstrated exemplary performance in handling multi-dimensional time series tasks. However, the above model fails to consider that noise and missing data in forecasting tasks are also key factors affecting the performance of the forecasting task.

This paper proposes a robust temporal nonnegative matrix factorization forecasting model (RTNMFFM) for multidimensional time series based on the $L_{2,1}$ norm [11]. It is a robust version of Temporal Nonnegative Matrix Factorization (TNMF) and focuses on multidimensional time series data forecasting. The overall contribution of our model is as follows:

- We propose RTNMFFM, a multi-dimensional time series matrix factorization method that can efficiently handle noise and missing values. RTNMFFM utilizes the $L_{2,1}$ norm as the loss function for non-negative matrix factorization to boost the model’s capacity to handle anomalous data and integrates an autoregressive (AR) regularizer [3] to capture the temporal correlation of the factor matrix. The model can automatically estimate missing values and make predictions.
- We propose a period smoothing penalty method using high-confidence time slices to improve the stability of predictions when data are severely missing.
- We propose an alternating optimization strategy applicable to RTNMFFM, using the Adam optimizer [12] to accelerate the training process. Experiments have shown that RTNMFFM provides state-of-the-art errors on noisy and missing data.

The rest of the paper is organized as follows: in Section 2, we briefly review the related work on multidimensional time series data forecasting. Section 3 describes our proposed RTNMFFM model in detail. Section 4 provides results from experiments based on several real-world datasets. This is followed by the conclusions and future work in Section 5.

## 2. Related Work

In this section, we will first introduce the time series forecasting model that deals with noise and missing data, and then we will concentrate on the time series forecasting model based on matrix factorization.

### 2.1. Time Series Forecasting Model That Can Handle Anomalous Data

To deal with noisy time series data, Singh et al. [13] discussed injecting Gaussian noise into time series data, applied Fourier analysis to filter the noise, and proposed a pattern modeling and recognition system (PMRS) to forecast noisy data. Laurinec et al. [14] proposed a density-based unsupervised ensemble learning method to improve forecasting accuracy for extremely fluctuating and noisy time series. Liu et al. [15] proposed an integrated three-phase model called adaptive noise reduction stacked autoencoder (ANR-SAE-VALSTM). The model uses ANR to filter out the noise and Long Short-Term Memory (LSTM) neural networks to forecast the data. Rasul et al. [16] proposed TimeGrad, a multi-dimensional probabilistic forecasting method using a diffusion probability model. The disadvantage is the high cost of model training.

For handling incomplete data, inputting data before forecasting is one class of methods. Sridevi et al. [17] proposed to use of ARLSimper, an autoregressive-based estimator of missing values, to repair the missing values and then forecast future data. In this way, the imputation algorithm’s effectiveness and performance indirectly influence the forecasting algorithm’s accuracy. On the other hand, some studies build forecasting models directly from missing data. Che et al. [18] proposed a decay mechanism integrated with a Gated Recursive Unit deep learning model (GRU-D). Bokde et al. [19] proposed a pre-processing algorithm. The method simultaneously forecasts and backcasts missing values for imputation by improving the Pattern Sequence Forecasting (PSF) algorithm.

However, as mentioned earlier, these methods, in which time series data with anomalous data are first preprocessed to remove missing values and outliers from the data and then predicted using certain time series prediction models, are prone to accumulating errors in the interpolation algorithm.

### 2.2. Forecasting Models Based on Matrix Factorization

Matrix factorization is a low-rank factorization model. Because of its ability to find latent factors in data, it is frequently used in clustering [20] and recommendation systems [21]. MF models such as non-negative matrix factorization (NMF) [22] and more general forms of tensor factorization (CP, Tucker) have been widely used for complex time-stamped events [9]. On time series data, matrix factorization is widely used for dimensionality reduction [23] and data imputation [24].

The default MF can only capture global low-rank features. For time series data, we want the decomposed matrix to maintain the temporal correlation of the original data. Early graph Laplacian regularization fails to establish negative correlations between time points [25]. To create a model of non-negative matrix factorization having temporal smoothness, Chen et al. [26]. construct the difference terms using Toeplitz matrices. Rao et al. [27] use a graph-based approach to introduce Laplacian regularization to deal with temporal correlation. The above regularization method maintains the temporal pattern, improves temporal smoothness, and shows better performance on interpolation tasks. However, these models cannot perform forecast missions. Yu et al. [3] developed a new regularization framework, and the proposed temporal regularized MF model (TRMF) can be applied to multidimensional time series data with missing values. TRMF can handle missing values while achieving forecasts through an autoregressive regularizer. Takeuchi et al. [28] obtained better forecasting performance by modeling spatiotemporal tensor data by introducing a spatial autoregressive regularizer. Sen et al. [29] proposed a global model combining MF and temporal convolutional network regularization. It can perform better with unnormalized data. Chen et al. [30] proposed a fully Bayesian matrix factorization (BTMF) framework to establish temporal correlation through vector autoregression (VAR) [31] models. Yet, BTMF’s use of the Gibbs sampling algorithm to increase the model’s robustness has a very high time cost, so it cannot be suitable for large datasets. Yang et al. [32] proposed to use the LSTM temporal regularizer matrix factorization model, but also Group Laplacian (GL) is used as a spatial regularizer to take advantage of the spatial correlation between sensors. More detailed comparisons between TRMF and other competitors can be found in Section 4.1.2.

However, the above framework mostly considers how to establish temporal correlation, ignoring the effects of noise, outliers, and missing values. Too many parameters in the model can also cause overfitting. To the best of our knowledge, the $L_{2,1}$ -norm has better robustness properties [33,34], and non-negative matrix factorization can reduce overfitting.

## 3. Proposed Method

### 3.1. Problem Description and Notation

In this paper, we assume that multidimensional time series data have some cross-dimensional correlation (like spatial correlation or common trends) and have data with non-negative constraints. In general, we organize the data collected by the M sensors with N time stamps as a matrix $Y\in R_{+}^{M\times N}$ . To denote the matrix, we utilize boldface and uppercase letters. (e.g., Y ), boldface and lowercase letters to denote column vectors (e.g., y ), and unbolded lowercase to denote scalars (e.g., *a*). We use the symbols listed in Table 1.

### 3.2. Constrained Non-Negative Matrix Factorization Algorithm

In this section, we will introduce the theory of the constrained non-negative matrix factorization algorithm, which is the basis of our proposed model.

Given a multidimensional non-negative time series data matrix $Y\in R_{+}^{M\times N}$ , the NMF can decompose the data matrix Y into an approximation of two K -dimensional low-rank non-negative matrices, such that Y≈UX ( K≪min(M,N) ), where $X\in R_{+}^{K\times N}$ is the latent temporal factor matrix and $U\in R_{+}^{M\times K}$ is the latent correlation factor matrix. Only under the sole non-negativity constraint, basic NMF will not obtain a unique solution [35]. So, in order for NMF to incorporate prior knowledge and adequately represent or reflect the problem’s relevant features, this can be achieved by adding regularization terms to U and/or X . It is easy to solve the non-negative matrix factorization with constraints by minimizing the objective function,

(1) $$\begin{array}{c} \min_{U,X}{∥Y-\mathrm{UX}∥}_{F}^{2}+\lambda_{U}J_{1}\left(U\right)+\lambda_{X}J_{2}\left(X\right),s.t.U\geq 0,X\geq 0, \end{array}$$

where $\lambda_{U}$ and $\lambda_{X}$ are the regularization parameters used to balance the goodness of fit and the constraint, $J_{1}\left(U\right)$ , and $J_{2}\left(X\right)$ are the penalty terms; they are utilized to enforce application-specific requirements. For example, $J_{1}\left(U\right)={∥U∥}_{F}^{2},J_{1}\left(X\right)={∥X∥}_{F}^{2}$ can be added to balance the goodness of fit and constrain the overall parameter size [35], where ${∥\;∥}_{F}$ is the Frobenius norm. The objective function (1) uses the squared Euclidean distance to calculate the difference between the original data matrix and the approximation UX .

### 3.3. Proposed Method

In this section, we propose RTNMFFM. It is a multidimensional time series forecasting model that effectively handles noise, outliers, and missing values. First, we will introduce how RTNMFFM handles anomalous data and establishes temporal dependencies in Section 3.3.1. Then, in Section 3.3.2, we will describe how RTNMFFM can alleviate the decrease in prediction accuracy when data are severely missing. In Section 3.3.3, we will describe in detail how the objective function is optimized. Finally, Section 3.3.4 describes how the model predicts.

#### 3.3.1. Time-Dependent Non-Negative Matrix Factorization Using the $L_{2,1}$ Norm

The objective function (1) measures the difference between the original matrix Y and the approximate UX in the form of the squared error. Due to the large mean square error of the anomalous data, the objective function is easily dominated by such data. RTNMFFM applies the $L_{2,1}$ norm to quantify the error between the original time series data and the approximation matrix. The $L_{2,1}$ norm of the matrix [34,36] Y is defined as

(2) $$\begin{array}{c} {∥Y∥}_{2,1}=\sum_{t=1}^{N}\sqrt{\sum_{i=1}^{M}Y_{it}^{2}}=\sum_{t=1}^{N}{∥y_{t}∥}_{2}. \end{array}$$

Note that it satisfies the triangle inequality as well as the three norm conditions and the $L_{2,1}$ norm is a legitimate norm [34]. RTNMFFM uses it as a measure of error.

(3) $$\begin{array}{cc} \min_{U,X}{∥Y-\mathrm{UX}∥}_{2,1}+\lambda_{U}{∥U∥}_{F}^{2}+ & \lambda_{X}{∥X∥}_{F}^{2}\\ s.t.U\geq 0,X\geq 0, \end{array}$$

where the first term is the robust formulation of the error function [37], ${∥\;∥}_{F}$ is the Frobenius norm for preventing overfitting [38] or guaranteeing strong convexity [3], and $\lambda_{U}$ and $\lambda_{X}$ are the regularization parameters. The robust formulation of the error function is equivalent to

(4) $${∥Y-\mathrm{UX}∥}_{2,1}=\sum_{t=1}^{N}\sqrt{\sum_{i=1}^{M}{\left(Y-\mathrm{UX}\right)}_{it}^{2}}=\sum_{t=1}^{N}{∥y_{t}-Ux_{t}∥}_{2}.$$

Equation (4) treats the $L_{2,1}$ norm as a measure of the loss of the reconstruction error. When there are outliers in the data, the data will show a fat-tailed distribution, and the Laplace distribution is one of the statistical methods to analyze the fat-tailed data, which has a low sensitivity to outliers and more robust features. Assume that the observation data vector $x_{t}$ is contaminated by noise $\varepsilon_{t}$ , obeying a Laplace distribution with mean zero.

(5) $$\begin{array}{c} y_{t}=\theta_{t}+\varepsilon_{t}, \end{array}$$

where $\theta_{t}$ is an unobservable truth value that can be considered as a point in a *K*-dimensional subspace ( K<M ), i.e.,

(6) $$\begin{array}{c} \theta_{t}=Ux_{t}, \end{array}$$

where $x_{t}$ is the projection of $y_{t}$ onto the subspace defined by the columns of U . Assuming that $\varepsilon_{t}$ obeys a Laplace distribution with zero mean and scale parameter *b*, thus $y_{t}∼La\left(\theta_{t},b\right)$ , and since in general, each vector $y_{t}$ in Y is independent, the probability distribution of $y_{t}$ conditional on $\theta_{t}$ is

(7) $$\begin{array}{c} p\left(y_{t}|\theta_{t}\right)∼\exp-\frac{|y_{t}-\theta_{t}|}{b}, \end{array}$$

the $L_{2,1}$ metric loss function has a rotational invariant property, while the pure $L_{1}$ loss function does not have such a desirable property. The literature [39] emphasizes the importance of rotational invariance in the context of learning algorithms. The reason the $L_{2,1}$ norm possesses rotational invariance is that its inner layer first solves for the $L_{2}$ norm of the vector, and rotational invariance is a fundamental property of Euclidean spaces with the $L_{2}$ norm [36]. Since the subspace is not uniquely determined before mapping the data to a lower-dimensional space, it is common to model the data with distributions that satisfy rotational invariance [36]. By the rotational invariance of the $L_{2,1}$ norm, the literature [36] generalizes the Laplace distribution to the rotationally invariant Laplace distribution (Equation (8)).

(8) $$\begin{array}{c} p\left(y_{t}|\theta_{t}\right)∼\exp-\frac{\left|\right|y_{t}-\theta_{t}{\left|\right|}_{2}}{b}, \end{array}$$

according to the strategy of maximizing the log-likelihood of the data, it can be obtained that

(9) $$\begin{array}{c} \max_{\theta_{t}}\log\prod_{t=1}^{N}p(y_{t}|\theta_{t})=\max_{\theta_{t}}-\frac{1}{b}\sum_{t=1}^{N}{\left||y_{t}-\theta_{t}|\right|}_{2}, \end{array}$$

Maximizing the data likelihood is equivalent to minimizing the summation part of Equation (9), so by replacing $\theta_{t}$ with ${\mathrm{Ux}}_{t}$ , we obtain Equation (10),

(10) $$\begin{array}{c} \min_{\theta_{t}}\sum_{t=1}^{N}{\left||y_{t}-\theta_{t}|\right|}_{2}=\min_{U,x_{t}}\sum_{t=1}^{N}{\left||y_{t}-Ux_{t}|\right|}_{2}=\min_{U,X}{\left||Y-\mathrm{UX}|\right|}_{2,1}. \end{array}$$

In Equation (10), assuming that the fitting error obeys a rotationally invariant Laplace distribution, the maximum likelihood problem is transformed into a non-negative matrix factorization problem with $L_{2,1}$ norm as the loss function by imposing the constraints U≥0 , X≥0 [37]. Typically, $L_{0}$ norms are ideal for eliminating outliers. The $L_{0}$ norm is the number of non-zero elements in a vector, and it can realize the sparsity constraint. However, solving the $L_{0}$ norm is an NP-hard problem and it is difficult to optimize. A common method is to solve an approximation problem for the $L_{0}$ norm [11]. Both the $L_{2,1}$ norm and the $L_{1}$ norm have the property that the $L_{0}$ norm makes the solution results sparse, so we believe that the $L_{2,1}$ norm can achieve the same robustness goal. In contrast to the loss function of the $L_{1}$ metric, the $L_{2,1}$ metric is convex and can be easily optimized [11,34].

**Figure 2:**
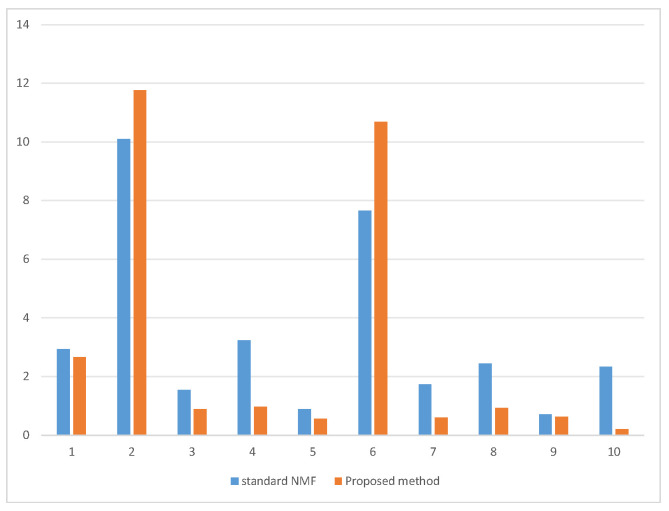
Plot of residue: $\left|X-\mathrm{UX}\right|$ by using standard NMF and the proposed method. Data points #2 and #6 are outliers.

From a computational point of view, the error for each data point in the robust formula is $∥y_{t}-{\mathrm{Ux}}_{t}{∥}_{2}$ , which prevents large errors due to outliers from dominating the objective function in the form of squared errors [34,37]. We use two-dimensional toy data to show the robustness of the $L_{2,1}$ metric loss function by generating 10 original two-dimensional data points, two of which are outliers. For each data point, we fit the original data points using the $L_{2,1}$ metric loss function and the standard non-negative matrix factorization, respectively. All data projections will be in the one-dimensional subspace. The residuals corresponding to each data point are shown in Figure 2, where the nonnegative matrix factorization of the $L_{2,1}$ metric loss function has a much smaller error compared to the standard NMF and is minimally affected by the two outliers. This robust approach keeps large errors caused by outliers from dominating the objective function by compressing the larger residual values. However, the model defined by the loss function in Equation (4) is not yet able to predict the data, and next, we will describe in detail how RTNMFFM constructs temporal correlations and predicts future data.

**Figure 3:**
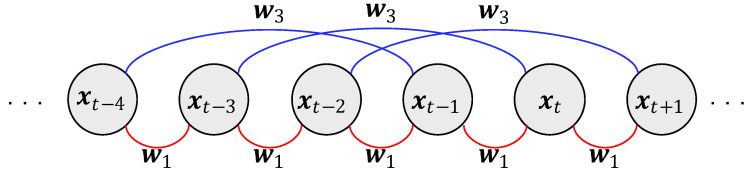
The temporal relationship with L={1,3} .

In general, the latent state of a particular timestamp may be related to the latent state of one or more timestamps preceding that timestamp. The relationship between the columns of the latent temporal factor matrix can be expressed as follows:

(11) $$\begin{array}{c} x_{t}\approx \sum_{l\in L}w_{l}⊙x_{t-l}, \end{array}$$

where L is a lag set storing the timing relationships between columns in X , $w_{l}$ is a vector of timing coefficients for $x_{t-l}$ , and all $w_{l}$ are combined into a timing coefficient matrix W . As in the timing structure shown in Figure 3, the state of each timestamp is then related to the state of the first and third timestamps preceding that timestamp. In this example, L={1,3} , $W=[w_{1},w_{3}]$ . In this example, any column $x_{t}$ of the matrix X is a combination of its first previous column $x_{t-1}$ and its third previous column $x_{t-3}$ in the following manner:

(12) $$\begin{array}{c} x_{t}\approx w_{1}⊙x_{t-1}+w_{3}⊙x_{t-3}. \end{array}$$

Based on the above temporal relationship between the columns of the latent temporal factor matrix X , by introducing an AR temporal regularizer [3] on top of the non-negative matrix factorization approach $J_{AR}\left(X|W,L,\lambda_{w}\right)$ , it learns in an autoregressive manner the matrix of temporal coefficients W , the matrix of latent correlation factor U , and the matrix of latent temporal factor X that are best adapted to the system without having to realize the artificial setting of the timing coefficients W .

The matrix factorization model that incorporates a temporal regularizer establishes a temporal relationship generation mechanism between the columns of X , and at the same time achieves the prediction of high-dimensional time series data. The AR regularizer is

(13) $$\begin{array}{c} J_{AR}\left(X|W,L,\lambda_{w}\right)=\sum_{t=l_{d}+1}^{N}{∥x_{t}-\sum_{l\in L}w_{l}⊙x_{t-l}∥}_{2}^{2}+\lambda_{w}{∥W∥}_{F}^{2}, \end{array}$$

where the first term in Equation (13), $L=\{l_{1},l_{2},\ldots ,l_{d}\}$ is a time lag set that indicates temporal correlation topology, the temporal structure indicated by the time lag set can be reflected in the matrix of latent temporal factors. $l_{d}$ is the maximum value of the time lag set ( $l_{d}=\max_{l\in L}\left(l\right)$ ), ⊙ is the element-wise product, $w_{l}$ is K×1 coefficient vector, and it is a learnable parameter representation of the autoregressive coefficients, and the last term is the regularization term of the $W=(w_{1},w_{2},\ldots ,w_{l_{d}})$ . The aim is to prevent overfitting. The set of time lags in AR regularizers can be discontinuous to flexibly embed seasonality [3].

Introducing constraint (13) into the loss function (3), we get

(14) $$\begin{array}{cc} \min_{U,X,W} & {∥Y-\mathrm{UX}∥}_{2,1}+\lambda_{U}{∥U∥}_{F}^{2}+\lambda_{X}{∥X∥}_{F}^{2}\\ & +\lambda_{AR}\sum_{t=l_{d}+1}^{N}{∥x_{t}-\sum_{l\in L}w_{l}⊙x_{t-l}∥}_{2}^{2}+\lambda_{w}{∥W∥}_{F}^{2}\\ & s.t.U\geq 0,X\geq 0 \end{array}$$

**Figure 4:**
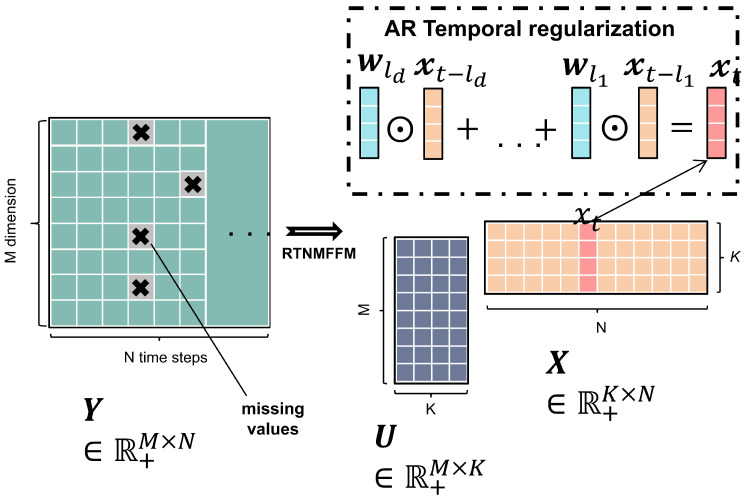
Illustration of RTNMFFM.

The illustration of RTNMFFM is shown in Figure 4, where RTNMFFM decomposes the data matrix Y on the left side into the dimensional feature matrix U and the temporal feature matrix X on the lower right-hand side, while the upper right side denotes the temporal dependence of RTNMFFM mining $x_{t}$ and its historical data through the autoregressive regularizer.

It is important to note that the model proposed in this paper can be viewed as a special kind of dynamic factor model (DMF) that

(15) $$\begin{array}{c} \begin{array}{cc} y_{t} & ={\mathrm{Ux}}_{t}+\varepsilon_{t},\\ x_{t} & =\sum_{l\in L}w_{l}⊙x_{t-l}, \end{array} \end{array}$$

Compared to TRMF, our proposed method is more robust and uses NMF rather than standard MF. The non-negative constraint of NMF on the latent temporal factor favors the generation of sparse encoding (more zero values). In learning autoregressive coefficients, fewer elements are involved in the training, thus reducing overfitting. Although more complex temporal regularizers have been studied and applied [29,32], RTNMFFM still chooses to capture the linear relationship of the AR model because NMF can only extract the linear structure of the data, and our study is more focused on robustness.

Where it is generally assumed that $\varepsilon_{t}$ to be serially uncorrelated (i.e., $\varepsilon_{t}∼$ i.i.d. N(0,Φ) , with Φ being a diagonal matrix).

The difference between our proposed method and Equation (15) is that first, our proposed model assumes that the noise $\varepsilon_{t}$ obeys a Laplace distribution. This assumption is more appropriate for describing the distribution of data in the presence of outliers. Another difference is that most of the existing studies [40,41,42] use principal component analysis (PCA) to obtain the sequence correlation, while our method uses NMF. Compare PCA, which uses a linear combination of all eigenbasis vectors to represent the data. Only the linear structure of the data can be extracted. In contrast, NMF represents data using combinations of different numbers and differently labeled basis vectors, so it can extract the multilinear structure of the data and has some nonlinear data processing capability.

#### 3.3.2. Periodic Smoothing Penalty for Severe Missing Values

**Figure 5:**
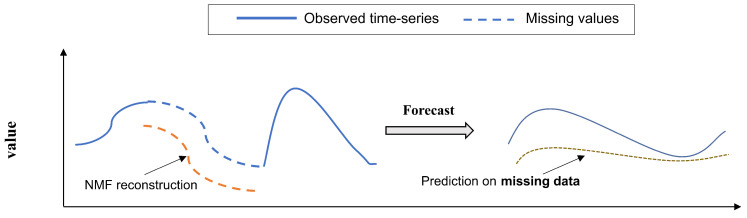
Example of the matrix factorization misalignment concerning the observed time series.

The percentage of missing data can be as high as 50% or more of the data collected by real sensors [10]. Severe missing values can result in inaccurate forecast values. A similar phenomenon was reported by Fernandes et al. [43], which they refer to as the “misalignment problem in matrix factorization with missing values”. This problem is illustrated in Figure 5: even though the nonnegative matrix factorization captures accurately the evolution of the time series in the missing gaps, the estimated range of values is far from the actual range of values. An intuitive observation of this problem is that the variation between consecutive timestamps is smooth, whereas this smoothness disappears when data are severely missing.

This unsmooth character carries over into the potential time factor matrix obtained by matrix factorization. When observations are missing, it causes the latent temporal factor matrix derived from the temporal regularization matrix factorization algorithm to lose its smoothing properties. This is manifested numerically in the form of lower numerical values of the latent temporal factor vectors for the time slices with missing data relative to the time slices with no missing time slices, and there is a misalignment of the vectors. The loss of smoothing the latent temporal factor matrix leads to overfitting, which prevents the autoregressive parameters from correctly representing the temporal relationships, and ultimately makes the prediction accuracy worse. Based on the above motivation, we need a way to improve the generalization ability, and our main goal is to create strategies for automatically smoothing these latent temporal factor vectors where the misalignment problem occurs.

The matrix factorization-based temporal regularization framework can be considered a specific example of a dynamic factor model (DFM) [44] that searches for linear links between high-dimensional observations and hidden states [30]. Imposing a smoothing penalty constraint on the latent variable can make the two variables similar, and we hope to reduce the effect of missing observations by adding some kind of smoothing penalty to the objective function to make the latent states of time slices with more missing values smoother.

Assuming a seasonal period of *T*, seasonality can make latent temporal factors X that are in the same in-phase similar, and in order to account for this, we need to rewrite the objective function to account for:

(16) $$\begin{array}{cc} \min_{U,X,W} & {∥Y-\mathrm{UX}∥}_{2,1}+\lambda_{U}{∥U∥}_{F}^{2}+\lambda_{X}{∥X∥}_{F}^{2}\\ & +\lambda_{AR}\sum_{t=l_{d}+1}^{N}{∥x_{t}-f_{w}\left(x_{t-1},x_{t-2},...,x_{t-l_{d}}\right)∥}_{2}^{2}+\lambda_{w}{∥W∥}_{F}^{2}\\ & s.t.U\geq 0,X\geq 0 \end{array}$$

where $f_{w}\left(\right)$ denotes the AR temporal regularizer. The temporal regularization can be formulated as follows:

(17) $$J_{AR}\left(X\right)=\lambda_{AR}\sum_{t=l_{d}+1}^{N}{∥x_{t}-x_{t}^{\prime }∥}_{2}^{2}$$

where $x_{t}^{\prime }$ is historical temporal feature vectors $x_{t-1},x_{t-2},\ldots ,x_{t-l_{d}}$ as input and make a forecast of $x_{t}^{\prime }=f_{w}\left(x_{t-1},x_{t-2},\ldots ,x_{t-l_{d}}\right)$ . The temporal regularizer can be viewed as $x_{t}$ doing smoothing with the predicted value $x_{t}^{\prime }$ of the autoregressive model, and this smoothing makes $x_{t}$ similar to $x_{t}^{\prime }$ , and due to seasonality, $x_{t}$ will be similar to both $x_{t-T}$ vectors. Based on this result, we envisioned whether we could do a smoothing penalty on the vectors of latent temporal factors that have lost their smoothing due to severe data missing versus the vectors of latent temporal factors with historical seasonality so that these factor vectors would regain their smoothing properties.

Since the missing values result in low numerical values of latent temporal factor vectors, we force smoothing of latent temporal factor vectors with low numerical values and latent temporal factor vectors with high numerical values, where the latent temporal factor vectors with high numerical values are in the same phase as the vectors with low values, by regularization penalties based on the presence of seasonality in the time series. For example, for any $x_{t}$ , the latent temporal factor vectors in the same phase as they are $x_{t-T}$ , $x_{t-2T}$ , … etc. The rationale is that latent temporal factor vectors that are in the same phase should be similar due to seasonality, as mentioned before. This is done to minimize the misalignment illustrated in Figure 5.

Based on this motivation, we propose to consider reducing the misalignment induced by missing values by applying a smoothing penalty to latent temporal factor vectors with lower numerical values. This smoothing penalty takes the form

(18) $$\begin{array}{c} J_{T}\left(X\right)=\lambda_{T}\sum_{t=l_{d}+1}^{N}{∥x_{t}-x_{\mathrm{ht}}∥}_{2}^{2} \end{array}$$

where $\lambda_{T}$ is the regularization parameter for the periodic smoothing penalty, $x_{t}$ is a vector of low numerical value latent temporal factors that need to be smoothed, and $x_{\mathrm{ht}}$ is a vector of latent temporal factors with smoother, higher numerical valued states of the potential state. This smoothing approach leads to two questions: (1) how to select the vectors that need to be smoothed? and (2) these selected vectors with low numerical values are smoothed with what kind of vectors $x_{\mathrm{ht}}$ ?

We propose to control these vectors and whether to use the periodic smoothing penalty based on the energy of the vectors in the temporal factor matrix $P_{t}$ at the moment *t*, where energy at the moment *t* of the time slice $x_{t}$ is

(19) $$\begin{array}{c} P_{t}=\sum_{i=0}^{K}x_{it}^{2}. \end{array}$$

We define $P_{\mathrm{mean}-T}$ as the average of the latent temporal factor vector energies of all latent temporal factors of vector $x_{t}$ up to moment *t* and with $x_{t}$ in the same phase. The form of $P_{\mathrm{mean}-T}$ is

(20) $$\begin{array}{c} P_{\mathrm{mean}-T}=\frac{1}{k+1}\sum_{t^{\prime }=t-k\cdot T}P_{t^{\prime }}\;,\left(l_{d}<t^{\prime }\leq t,k=0,1,2,\ldots ,k\cdot T<t\right). \end{array}$$

To prevent overfitting, the periodic smoothing penalty is not applied to all time slices and is only used when the energy $P_{t}$ of the current latent temporal factor is smaller than $P_{\mathrm{mean}-T}$ , which is consistent with the motivation that we mentioned before, i.e., we only need to smooth potential space-time factor vectors that have lower values. The energy $P_{t}$ of the potential temporal factor vector with lower values will be smaller. For example, suppose the seasonality is *T* = 3 and the time lag set is L={1,3} . We need to compute $P_{\mathrm{mean}-T}$ for $x_{10}$ . The steps are to first calculate the energies $P_{7}$ and $P_{4}$ for $x_{7}$ and $x_{4}$ . After that, find their average value $P_{\mathrm{mean}-T}=\left(P_{7}+P_{4}\right)/2$ . Finally, when $P_{10}$ is smaller than $P_{\mathrm{mean}-T}$ , a periodic smoothing penalty on $x_{10}$ is required. If $P_{10}$ is bigger than $P_{\mathrm{mean}-T}$ , we will force the regularization parameter $\lambda_{T}=0$ to indicate that x10 does not participate in the periodic smoothing penalty. This judgment prevents higher-energy time slices from participating in the periodic smoothing penalty and prevents normal potential time factor vectors from losing certain features that should be present due to overfitting previous time vectors instead. We utilize the property that the seasonality of the time series leads to similar values of the latent temporal factor vectors, assuming that the current latent temporal factor vector $x_{t}$ is too low due to missing data, the energy of this vector will be lower than the average energy of the historical vectors of the same phase, and we pick out vectors that need to be smoothed by this method.

For the second problem, we define the high-confidence latent temporal vector $x_{\mathrm{ht}}$ of $x_{t}$ to be the latent temporal vector that is historically in the same phase as $x_{t}$ and has the highest energy. The reason for this is that latent temporal factor vectors with lower values need to be smoothed with latent temporal vectors with higher values, which should have relatively higher energies. Secondly, due to seasonality, latent temporal factors that are in the same phase should be similar. For example, if $x_{10}$ needs to perform a periodic smoothing penalty, we need to find the maximum energy latent temporal factor vector in $x_{7}$ and $x_{4}$ and use it as the high-confidence latent temporal vector $x_{\mathrm{ht}}$ for $x_{10}$ .

**Figure 6:**
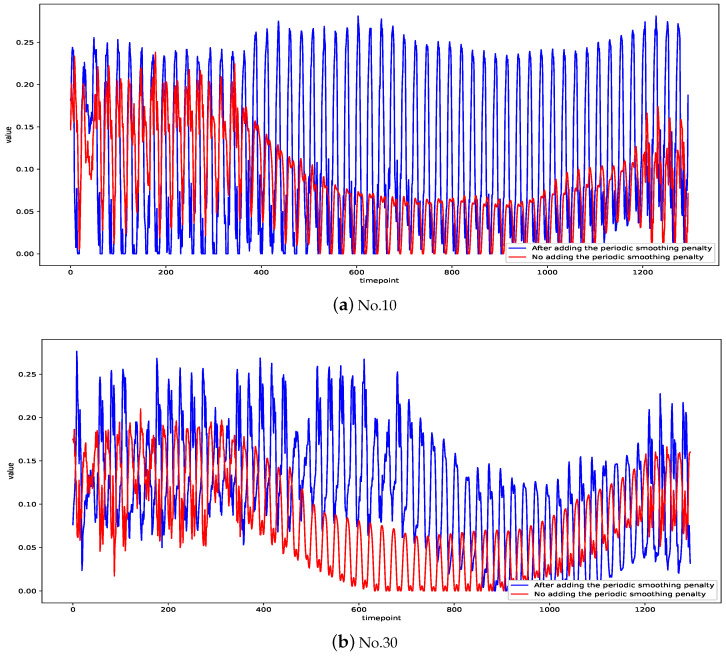
Results with and without periodic smoothing penalty added.

We give an intuitive explanation of the results of the latent temporal factor matrix decomposed by the temporal regularization matrix factorization after adding the periodic smoothing penalty we proposed, as compared to the latent temporal factor matrix without adding the periodic smoothing penalty, using an example. We experimented with temporal regularization matrix factorization on the Guangzhou Urban Traffic Speed dataset with a data size of 214 × 1380, and we let all the data from *t* = 360 to *t* = 1200 as missing. We set the rank of the low-rank matrix at *K* = 40. In Figure 6, the blue line is the result after adding the periodic smoothing penalty, and the red line is not added. It can be seen that the latent temporal factors are smoother with the added periodic smoothing penalty, which is very obvious at *t* = 360 to *t* = 1200. This shows that our proposed periodic smoothing penalty can make the latent temporal factor matrix smoother, more conducive to the learning of the parameters in the temporal regularization term, and more conducive to the final prediction.

The loss function with the addition of the periodic smoothing penalty is

(21) $$\begin{array}{cc} \min_{U,X,W} & {∥Y-\mathrm{UX}∥}_{2,1}+\lambda_{U}{∥U∥}_{F}^{2}+\lambda_{X}{∥X∥}_{F}^{2}\\ & +\lambda_{AR}\sum_{t=l_{d}+1}^{N}{∥x_{t}-\sum_{l\in L}w_{l}⊙x_{t-l}∥}_{2}^{2}\\ & +\lambda_{T}\sum_{t=l_{d}+1}^{N}{∥x_{t}-x_{ht}∥}_{2}^{2}+\lambda_{w}{∥W∥}_{F}^{2}\\ s.t.U & \geq 0,X\geq 0. \end{array}$$

The objective function in Equation (21) focuses on the severely missing data. For lower-energy time slices, RTNMF actively utilizes high confidence slices, while for higher-energy time slices, no smoothing is performed to prevent overfitting.

#### 3.3.3. Optimization

To minimize the objective function in Equation (21), we propose a gradient descent-based algorithm. The optimization of the $L_{2,1}$ norm can be found in [11,37]. RTNMFFM will alternate the optimization of each factor matrix; it updates just one-factor matrix at a time while fixing all other factor matrices. We use the Adam optimizer [12], which has been successful in deep learning, to accelerate training. Suppose we can update the pertinent parameters as follows at iteration *p*.

**Updates for latent correlation factor matrix U .** Find the partial derivative of matrix U .

(22) $$\nabla_{U}F\left(U^{\left(p\right)},X^{\left(p\right)},W^{\left(p\right)}\right)=-2\left(Y-U^{\left(p\right)}X^{\left(p\right)}\right){{\mathrm{DX}}^{\left(p\right)}}^{⊤}+2\lambda_{U}{U^{\left(p\right)}}^{⊤},$$

where ⊤ is the transpose of a matrix or vector and ***D*** is a diagonal matrix with the diagonal elements given by

(23) $$\begin{array}{c} D_{tt}=1/\sqrt{\sum_{i=1}^{M}{\left(Y-\mathrm{UX}\right)}_{it}^{2}}=1/{∥y_{t}-{\mathrm{Ux}}_{t}∥}_{2}. \end{array}$$

Update the matrix $U^{\left(p+1\right)}$

(24) $$\begin{array}{c} U^{\left(p+1\right)}=P_{+}(U^{\left(p\right)}-\alpha \nabla_{U}F\left(U^{\left(p\right)},X^{\left(p\right)},W^{\left(p\right)}\right), \end{array}$$

where α is the learning rate and $P_{+}$ denotes the projection operator that forces all elements of it to be projected onto a non-negative semi-axis; this takes the form of Equation (25).

(25) $$P_{+}\left(x\right)=\max\left\{x,0\right\}$$

**Updates for latent temporal factor matrix X .** The gradient of the solver matrix X is divided into two parts. First, calculate the gradient of the first term in Equation (21):

(26) $$\begin{array}{c} -2U^{{\left(p+1\right)}^{⊤}}\left(Y-U^{\left(p+1\right)}X^{\left(p\right)}\right)D+2\lambda_{X}X^{\left(p\right)}, \end{array}$$

where the matrix D has the same form as Equation (23). Second, for vectors $x_{t}$ t=1,2,…, *N*, calculate the gradient of the AR regularization term and the periodic smoothing penalty term by column.

When $t>l_{d}$ :

(27) $$\begin{array}{c} \begin{array}{c} \lambda_{T}\left(x_{t}^{\left(p\right)}-x_{ht}\right)+2\lambda_{AR}\left[x_{t}^{\left(p\right)}-\sum_{l\in L}w_{l}^{\left(p\right)}⊙x_{t-l}^{\left(p\right)}+2{w_{l}^{\left(p\right)}}^{2}⊙x_{t}^{\left(p\right)}\right.\\ \left.-\sum_{\underset{t+l<N}{l\in L,}}w_{l}^{\left(p\right)}⊙{\left(x_{t+l}^{\left(p\right)}-\sum_{l^{\prime }\in L-\left\{l\right\}}w_{l^{\prime }}^{\left(p\right)}⊙x_{t+l^{\prime }-l}^{\left(p\right)}\right)}^{⊤}\right], \end{array} \end{array}$$

combine all column vectors with Equation (27) into a matrix and add Equation (26) in $\nabla_{X}F\left(U^{\left(p+1\right)},X^{\left(p\right)},W^{\left(p\right)}\right)$ to update in matrix form

(28) $$\begin{array}{c} X^{\left(p+1\right)}=P_{+}(X^{\left(p\right)}-\alpha \nabla_{X}F\left(U^{\left(p+1\right)},X^{\left(p\right)},W^{\left(p\right)}\right). \end{array}$$

**Updates for AR regularizer parameters W .** For vectors $w_{i}$ , $i=1,2,\ldots ,l_{d}$ :

(29) $$\begin{array}{cc} & \nabla_{w}F\left(U^{\left(p+1\right)},X^{\left(p+1\right)},w_{i}^{\left(p\right)}\right)=\\ & \lambda_{AR}\sum_{t=l_{d}+1}[-2\left(x_{t}^{\left(p+1\right)}-\sum_{l\in L-\left\{i\right\}}w_{i}^{\left(p\right)}⊙x_{t-i}^{\left(p+1\right)}\right)⊙x_{t-i}^{\left(p+1\right)}\\ & +2diag\left(x_{t-i}^{\left(p+1\right)}⊙x_{t-i}^{\left(p+1\right)}\right)w_{i}^{\left(p\right)}+\lambda_{W}w_{i}^{\left(p\right)}], \end{array}$$

combine all column vectors into a matrix $\nabla_{W}F\left(U^{\left(p+1\right)},X^{\left(p+1\right)},W^{\left(p\right)}\right)$ to update in matrix form

(30) $$\begin{array}{c} W^{\left(p+1\right)}=W^{\left(p\right)}-\alpha \nabla_{W}F\left(U^{\left(p+1\right)},X^{\left(p+1\right)},W^{\left(p\right)}\right). \end{array}$$

**Overall training.** Algorithm 1 describes the entire training process of RTNMFFM. First, initialize each factor with a non-negative constraint. We update sequentially each factor matrix for each iteration while using the Adam optimizer [12] to accelerate training. We will select the model with the lowest error on the validation set as the final model.
**Algorithm 1** Training RTNMFFM
**Input:** Observed data matrix $Y\in R_{+}^{M\times N}$ , rank *K*, lag set L , learning rate α , regularization parameter $\lambda_{U}$ , $\lambda_{X}$ , $\lambda_{AR}$ and $\lambda_{T}$ , number of iterations p=0 , validation set $Y^{\prime }$ ;**Output:** Final values for U , X , W with the lowest error on the validation;1:Initialize all matrices $U^{\left(0\right)}$ , $X^{\left(0\right)}$ , $W^{\left(0\right)}$ ;2:**repeat**3:    Update $U^{\left(p+1\right)}=P_{+}(U^{\left(p\right)}-\alpha \nabla_{U}F\left(U^{\left(p\right)},X^{\left(p\right)},W^{\left(p\right)}\right)$ using Adam;4:    Calculate high-confidence time slices and determine whether period smoothing is needed;5:    Update $X^{\left(p+1\right)}=P_{+}(X^{\left(p\right)}-\alpha \nabla_{X}F\left(U^{\left(p+1\right)},X^{\left(p\right)},W^{\left(p\right)}\right)$ using Adam;6:    Update $W^{\left(p+1\right)}=P_{+}(W^{\left(p\right)}-\alpha \nabla_{W}F\left(U^{\left(p+1\right)},X^{\left(p+1\right)},W^{\left(p\right)}\right)$ using Adam;7:**until** convergence criterion is met;8:**return** The matrix U , X , W with the lowest error on the validation set $Y^{\prime }$ .


#### 3.3.4. Forecasting

For RTNMFFM forecasting, given matrices U , X , and the time regularizer parameter W , the latent time factor vector at the moment t+1 in the future is forecasted by the autoregressive model in a single step

(31) $$\begin{array}{c} {\hat{x}}_{t+1}=\sum_{l\in L}w_{l}⊙x_{t+1-l}. \end{array}$$

**Figure 7:**
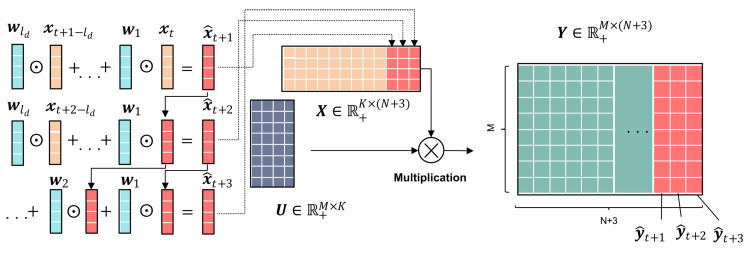
Illustration of the one-step rolling forecasting scheme.

The model is based on one-step forecasting and performs multi-step forecasting by recursively reusing the predicted values as input and then estimating observed time series data with ${\hat{y}}_{t+1}=U{\hat{x}}_{t+1}$ . Figure 7 illustrates how the model makes multi-step forecasts.

## 4. Experiments

To demonstrate the performance of the proposed model, in this section, we experiment with five datasets. We set up multiple noise and missing forms to fully validate the model. All experiments are performed on a machine equipped with Intel i7-10700. The software environment is Python 3.8, Numpy 1.22.3.

### 4.1. Experimental Settings

#### 4.1.1. Datasets

- *Dataset (G): Guangzhou urban traffic speed (https://zenodo.org/record/1205229#.ZE9_kM5BxPY, accessed on 18 January 2024).* This dataset tracks the speed of traffic on 214 road segments over the course of two months at a 10-minute resolution. We change the data to reflect hourly speed by aggregating blocks of six columns and organizing the raw data set into a time series matrix of 214 × 1380 and there are 1.3 percent missing values.
- *Dataset (H): Hangzhou metro passenger flow. (https://tianchi.aliyun.com/competition/entrance/231708/information accessed on 18 January 2024),* With a 10-minute resolution, this dataset collects inbound passenger flows from 80 subway stations in Hangzhou, China. We ignore the timeframe 0:00 a.m.–6:00 a.m. and structure the dataset into a matrix of 80 × 2700 and there are no missing data.
- *Dataset (P): Pacific surface temperature. (http://iridl.ldeo.columbia.edu/SOURCES/.CAC/, accessed on 18 January 2024)* This data set includes measurements of the Pacific Ocean’s monthly sea surface temperature taken over 396 consecutive months between January 1970 and December 2002. The size of the matrix is 2520 × 396 and there are no missing data.
- *Dataset (C): California freeways occupancy rate (https://archive.ics.uci.edu/ml/datasets/PEMS-SF, accessed on 18 January 2024).* These data comes from the California Department of Transportation. These data record lane occupancy rates on San Francisco Bay Area freeways. We combine the 10-minute sampling data into hourly data and confine our research to the last 2736 timestamps to organize the data into a matrix of 963 × 2736 and there are no missing data.
- *Dataset (E): Electricity load data (https://archive.ics.uci.edu/ml/datasets/ElectricityLoadDiagrams20112014, accessed on 18 January 2024).* A total of 370 clients’ electricity consumption data with a sampling frequency of 15 min. We use the last 10272 timestamps of electrical data to organize the data into a matrix of 370 × 10272 and there are no missing data.

#### 4.1.2. Metrics

We use Normalized Deviation (ND) and Root Mean Squared Error (RMSE) to evaluate performance, defined as follows.

$$\mathrm{ND}=\frac{\sum_{i,t\in \Omega }\left|Y_{it}-{\hat{Y}}_{it}\right|}{\sum_{i,t\in \Omega }\left|Y_{it}\right|},\mathrm{RMSE}=\sqrt{\frac{1}{\left|\Omega \right|}\sum_{i,t\in \Omega }{\left(Y_{it}-{\hat{Y}}_{it}\right)}^{2}}$$

where Ω indicates the test set, $Y_{it}$ indicates a forecasted entry with index (i,t) , and ${\hat{Y}}_{it}$ is the forecast results.

#### 4.1.3. Competitors

We compare RTNMFFM with the following competitors.

- TRMF [3]: A temporally regularized matrix decomposition method (https://github.com/xinychen/transdim, accessed on 18 January 2024). We rewrite this as a gradient-based version. Framework for predicting data with AR.
- ARNMF [4]: Non-negative constrained version of TRMF. Our purpose in using this method as a baseline is to verify whether NMF is the main reason for the better performance achieved by RTNMFFM. Framework for predicting data with AR.
- SVD-AR(1) [3]: The rank-*K* approximation of $Y={\mathrm{USV}}^{⊤}$ is first obtained by singular value decomposition (SVD). After setting F=US and $X=V^{⊤}$ , a *K*-dimensional AR(1) is learned on X for forecasting.
- BTMF [30]: A Bayesian Temporal Matrix Factorization (BTMF) framework by incorporating a VAR layer into Bayesian probabilistic matrix factorization algorithms (https://github.com/xinychen/transdim, accessed on 18 January 2024). The framework is predicted by using the observation at the corresponding position in the previous time period as the predicted value. It can be considered as a seasonal naive forecast method after the data are complemented with high accuracy.
- LSTM-ReMF [32]: LSTM-ReMF framework by incorporating an LSTM layer into matrix factorization algorithms (https://github.com/Vadermit/TransPAI, accessed on 18 January 2024). Framework for predicting data with LSTM.

#### 4.1.4. Hyper-Parameter

The hyper-parameters used are in Table 2. The last few timestamps in the data are used as the test and validation sets, the lengths of which are shown in Table 2. For matrix factorization models using AR as a regularizer, all regularization parameters and learning rates are selected from the validation set, using the same rank-*K*, time lag set, and size of the forecasting window. For all algorithms, use a grid search to select all regularization parameters and learning rates, and set the hyperparameter set for which the model obtains the minimum RMSE on all sub-datasets as the final hyperparameters. All gradient-based algorithms are trained using Adam [12] acceleration. For BTMF and LSTM-ReMF, we use the hyperparameters suggested in the original paper. For all models, the data were min-max normalized before training the models, and we chose to have the lowest ND in the validation set as the final model.

### 4.2. Forecasting Accuracy

In Table 3, we compared the forecasts of RTNMFFM with those of competitors on the raw data. The results of all experiments are given by “ND/RMSE”. RTNMFFM(0) indicates that RTNMFFM does not use a periodic smoothing penalty.

RTNMFFM has almost obtained the best results, and we consider that the distribution of residuals on some datasets is the root cause of the poor performance of RTNMFFM. If the residuals obey a Gaussian distribution, their maximum likelihood problem can be converted to a standard NMF problem, which may be the reason why models based on standard NMF can take the lead in Data (H) and Data (E). Even so, the method proposed in this paper still gives sub-optimal results on both datasets. A comparison of the run times of the models can be found in Appendix A. Moreover, to verify the effect of noise on RTNMFFM, we added Gaussian noise and Laplace noise to all the data in Section 4.2.1. In Section 4.2.2, we test the forecasting performance of the proposed model under multiple missing modes.

#### 4.2.1. Forecasting Performance in Noisy Data

To verify the validity of the model under different background noises, we added Gaussian white noise (mean is μ=0 , variance is σ=1 ) and Laplace noise (position parameter μ=0 and scale parameter b=1 ) to each dataset to generate signal-to-noise ratios of 5, 2, 0, −2, and −5. The definition of the signal-to-noise ratio is

(32) $$\begin{array}{c} \mathrm{SNR}=10\log_{10}\frac{P_{s}}{P_{n}}, \end{array}$$

where $P_{s}$ is the power of the signal and $P_{n}$ is the power of the noise. The data we obtained are discrete, so for one piece of time series data $y_{i}$ of the observation matrix Y , the signal power is

(33) $$\begin{array}{c} P_{s}=\frac{1}{n}\sum_{t=1}^{N}y_{it}. \end{array}$$

To add noise of a certain signal-to-noise ratio to a signal, the method is to first compute the average power of the original signal, then compute the noise power by Equation (32). Finally, generate noise that obeys the Laplace distribution or the standard normal distribution and increase it by $\sqrt{P_{n}}$ times. These added noises are only present in the training set and have no effect on the valid set and the test sets. In order to maintain the distribution of noise added to the data, we relaxed the non-negativity of the data and subjected the data to min-max normalization before training. It is important to note that here RTNMFFM does not use the period smoothing penalty (i.e., $\lambda_{T}=0$ ).

**Figure 8:**
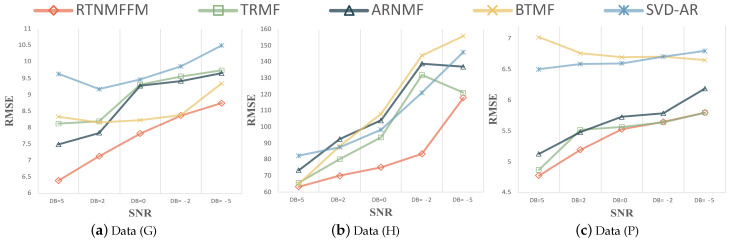

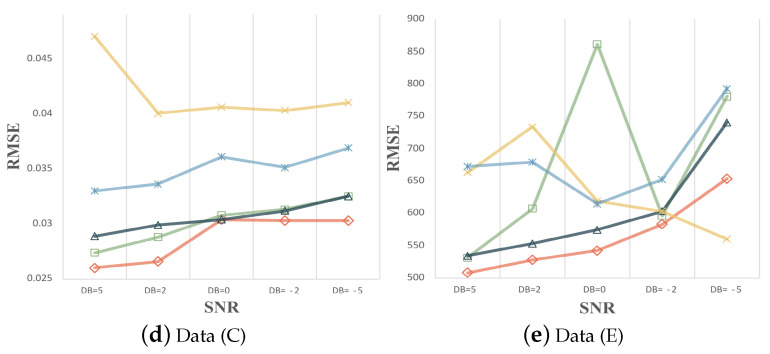
Test RMSE of RTNMFFM and competitors for varying SNR with Gaussian noise.

**Figure 9:**
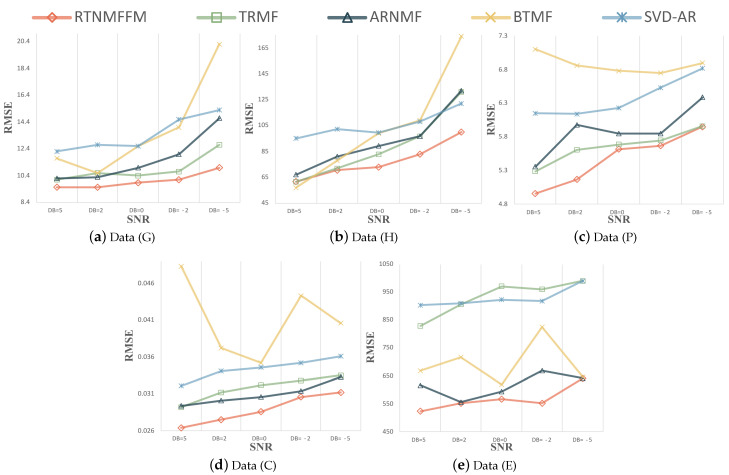
Test RMSE of RTNMFFM and competitors for varying SNR with Laplacian noise.

**Forecasting results and Analysis.** The forecasting performance of the model after adding Gaussian noise and Laplace noise to the five datasets is given in Figure 8 and Figure 9. All errors are calculated after undoing the scaling. The RTNMFFM model does not impose a periodic smoothing penalty.

RTNMFFM shows competitive forecasting results in these matrix-based decomposition models. When the source of real noise is particularly complex, Gaussian noise may be considered the best simulation of real noise. In Figure 8, RTNMFFM has an absolute advantage in forecasting performance in Gaussian noise. For datasets (G) and (H), the forecasting results of RTNMFFM were more significant compared to other competitors. A possible reason is that the length of these two datasets is smaller, and RTNMFFM is better able to learn temporal correlation from the limited information under noise. Comparing the results of RTNMFFM and ARNMF, the $L_{2,1}$ norm plays a dominant role in handling the noise. In addition, to more fully validate the RTNMFFM, the performance of the model in Laplace noise is shown in Figure 9. As we have seen, RTNMFFM can still learn the temporal dependencies from noisy data, and it can mitigate the effects of noise and make better forecasts.

#### 4.2.2. Forecasting Performance in Missing Data

In this section, we validate the effectiveness of the proposed model in making forecasts under various data loss scenarios. For one type of random point-wise missing (RM), we randomly removed certain observations and modeled random missingness with a value of zero. The other is continuous missing (CM), where data is missing for a sustained period of time. Continuously missing corresponds to a situation where the sensor has a certain probability of failing on a certain day. The missing setup method is taken from the literature [32], where continuous missing exists for multiple intervals and all sensors will have missing data. We tested the forecasting accuracy of each model at 60%, 50%, 40%, and 20% missing proportions. These added missing data are only present in the training set and have no effect on the valid set and test sets. We apply the proposed period smoothing penalty to TRMF as well to test its scalability (named TRMF(1)).

**Forecasting results and Analysis.**Table 4 and Table 5 demonstrate the forecasting performance of RTNMFFM and other baseline models for datasets (G), (H), (P), (C), and (E). For random missing values, RTNMFFM leads to prediction accuracy across the board. RTNMFFM works well for two categories of missing data when more than 50% of the data is missing. For continuous missing values, except in dataset (H), RTNMFFM exhibits superior predictive accuracy even in the presence of persistent missing data. Comparing RTNMFFM with BTMF, BTMF is as robust in the dataset (H), and its excellent performance stems from the Bayesian probabilistic matrix factorization algorithms.

Comparing RTNMFFM with its version without the period smoothing penalty (RTNMFFM(0)), the forecasting results show that our proposed period smoothing penalty can improve the prediction ability to some extent when severe data are missing.

From the forecasting results, it can be seen that RTNMFFM(0) can also obtain good results in some cases. This model based on the $L_{2,1}$ norm measure of matrix factorization error is also known as indirect sparse matrix factorization with $L_{2,1}$ norm [33], which indirectly optimizes the upper bound of the F-norm function in a way that is the reason for its ability to find more effective information from sparse data and obtain more accurate predictions. In order to compare the variability of the predictions, we also compared the results of the Diebold–Mariano test [45] for RTNMFFM and the best alternative model, and the conclusions are shown in Appendix B.

Comparing TRMF and TRMF(1), TRMF(1) has better forecasting accuracy, which proves the scalability and effectiveness of the period smoothing penalty proposed in this paper. Similar to the previous conclusions, the model after adding the period smoothing penalty is more effective when the amount of missing data is high.

The forecasting results of RTNMFFM are more significant on datasets (P), (C), and (E). For datasets (C) and (E), they have more sampling cycles, RTNMFFM can select high-confidence time slices from more samples to apply the period smoothing penalty, and the number of time series entries in the dataset (P) is much larger than the timestamps. The above features may be the reason why RTNMFFM is more competitive. Even on the poorly performing dataset (G), RTNMFFM remains optimal even at more than 50% missing values. However, for 20% of the missing data, RTNMFFM performs slightly worse. The biggest reason is that overfitting is generated. Therefore, we suggest using the period smoothing penalty when there are more missing values or performing a period smoothing penalty only in the initial phase of model learning.

**Figure 10:**
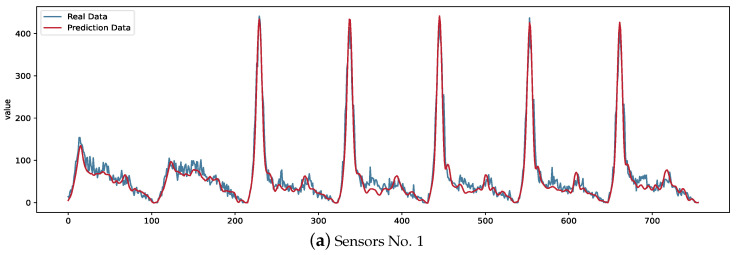

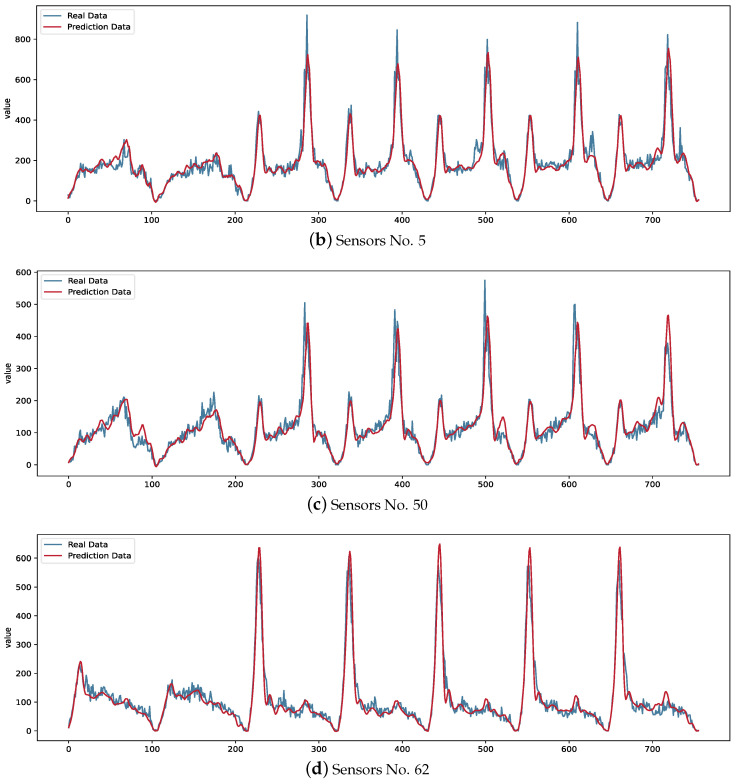
Predicted metro passenger flow (red curves) of RTNMFFM at 60% RM missing.

Next, we will show the forecast visualization of RTNMFFM on missing data. Figure 10 shows the prediction visualization of RTNMFFM on the Hangzhou metro passenger flow dataset, with 60% of the data randomly missing in the figure; the red line is the predicted value, and the blue line is the true value. As shown in the figure, the red and blue lines match each other for drastic short-term changes and long-term fluctuations, demonstrating our method’s effectiveness under severe missingness.

## 5. Conclusions and Future Work

This paper proposes a robust forecasting model for multidimensional time series data. We integrate the $L_{2,1}$ norm and the AR regularizer into the non-negative matrix factorization algorithm. This combination can better establish the temporal correlation of multidimensional time series data when there is noise and missing values in the data. Also, the proposed period smoothing penalty for RTNMFFM improves the accuracy of the prediction task on incomplete data. RTNMFFM provides a powerful tool for multidimensional time series prediction tasks, and we have examined the model on several real-world time series matrices. RTNMFFM has shown superior performance compared to other baseline models. There are several directions for future research to explore. First, an important reason why time series data is difficult to predict is data drift, i.e., the data distribution may change as time evolves, and an online learning framework is needed to correct the prediction results. Secondly, our model can establish trends and seasonality well, but it is not easy to establish cyclicality. Finally, our model can only establish linear correlations, and the modeling of nonlinear temporal correlations based on deep learning matrix decomposition and diffusion models is the focus of future research.

## Figures and Tables

**Table 1:** Table of symbols.

Symbol	Definition
Y	Observation data matrix $\in R_{+}^{M\times N}$
U	Latent correlation factors matrix/Loading matrix $\in R_{+}^{M\times K}$
X	Latent temporal factors matrix $\in R_{+}^{K\times N}$
${∥X∥}_{F}$	Frobenius norm of matrix X
${∥X∥}_{2,1}$	$L_{2,1}$ norm of matrix X
${∥x∥}_{2}$	$L_{2}$ norm of vector x
⊙	Element-wise multiplication of vectors, Hadamard product of matrix
L	Time lag set that indicates temporal correlation topology
$l_{d}$	Maximum value of time lag set
⊤	Transpose matrix or vector
$u_{j}^{⊤}$	The j th row of the matrix U
$x_{t}$	The t th column of the matrix X
Ω	The set of observed entries
$\lambda_{AR}$ , $\lambda_{w}$	Regularization parameter

**Table 2:** Default hyper-parameter setting, V-length/T-length indicates the number of validation set/test set timestamps.

Dataset	Time Lags Set- L	V-Length	T-Length	Rank-K
Data (G)	[1, …, 24] ∪ [ 7×24 , …, 7×24+24 ]	84	84	40
Data (H)	[1, …, 12] ∪ [756, …, 768]	378	378	20
Data (P)	[1, …, 3] ∪ [12, …, 15] ∪ [24, …, 27]	6	6	60
Data (C)	[1, …, 24] ∪ 7×24 , …, 7×24+24 ]	168	168	60
Data (E)	[1, …, 12] ∪ [ 7×96 , …, 7×96+12 ]	336	336	40

**Table 3:** Performance comparison (in ND/RMSE) for prediction tasks on data sets (G), (H), (P), (C), and (E).

Model	Data (G)	Data (H)	Data (P)	Data (C)	Data (E)
RTNMFFM	0.116/0.112	0.167/0.0557	0.109/0.161	0.257/0.071	0.195/0.113
RTNMFFM(0)	0.129/0.113	0.170/0.0567	0.116/0.194	0.274/0.073	0.202/0.116
TRMF	0.120/0.123	0.204/0.0681	0.107/0.166	0.299/0.077	0.212/0.129
ARNMF	0.118/0.120	0.164/0.0545	0.107/0.167	0.314/0.078	0.186/0.111
SVD-AR	0.142/0.136	0.211/0.0746	0.121/0.187	0.412/0.101	0.229/0.134
BTMF	0.134/0.115	0.181/0.0582	0.175/0.216	0.425/0.103	0.219/0.140
LSTM-ReMF	0.275/0.201	0.357/0.109	0.165/0.192	0.473/0.114	0.299/0.173

The best results are underlined.

**Table 4:** Performance comparison (ND/RMSE) for forecasting tasks with random missing.

Data	Missing	RTNMFFM	RTNMFFM(0)	TRMF(1)	TRMF	ARNMF	BTMF
(G)	60%RM	0.112/0.108	0.117/0.112	0.115/0.112	0.115/0.113	0.115/0.112	0.151/0.132
	50%RM	0.110/0.109	0.112/0.107	0.115/0.114	0.117/0.115	0.115/0.114	0.150/0.136
	40%RM	0.119/0.113	0.116/0.106	0.119/0.117	0.118/0.115	0.119/0.117	0.143/0.132
	20%RM	0.117/0.112	0.115/0.110	0.119/0.118	0.120/0.118	0.119/0.118	0.150/0.136
(H)	60%RM	0.176/0.059	0.184/0.062	0.192/0.064	0.201/0.066	0.192/0.064	0.183/0.059
	50%RM	0.167/0.056	0.174/0.058	0.178/0.060	0.198/0.064	0.178/0.060	0.180/0.058
	40%RM	0.169/0.056	0.169/0.057	0.169/0.056	0.166/0.054	0.168/0.056	0.184/0.059
	20%RM	0.168/0.056	0.165/0.055	0.173/0.057	0.193/0.060	0.173/0.057	0.183/0.059
(P)	60%RM	0.048/0.058	0.054/0.063	0.107/0.166	0.110/0.162	0.107/0.166	0.202/0.240
	50%RM	0.050/0.059	0.060/0.061	0.105/0.166	0.119/0.171	0.105/0.166	0.206/0.251
	40%RM	0.041/0.049	0.042/0.050	0.112/0.163	0.115/0.167	0.112/0.163	0.200/0.242
	20%RM	0.076/0.081	0.059/0.065	0.105/0.164	0.109/0.162	0.105/0.164	0.198/0.254
(C)	60%RM	0.260/0.071	0.261/0.072	0.332/0.082	0.308/0.077	0.332/0.082	0.396/0.100
	50%RM	0.277/0.072	0.274/0.073	0.296/0.077	0.298/0.076	0.296/0.077	0.603/0.134
	40%RM	0.278/0.074	0.284/0.077	0.304/0.076	0.301/0.077	0.304/0.076	0.419/0.103
	20%RM	0.272/0.071	0.261/0.071	0.283/0.074	0.295/0.076	0.283/0.074	0.392/0.098
(E)	60%RM	0.174/0.104	0.199/0.114	0.196/0.114	0.219/0.132	0.196/0.114	0.190/0.123
	50%RM	0.179/0.105	0.208/0.118	0.180/0.107	0.216/0.128	0.180/0.107	0.192/0.123
	40%RM	0.169/0.102	0.170/0.103	0.191/0.111	0.222/0.134	0.191/0.111	0.194/0.124
	20%RM	0.190/0.111	0.188/0.117	0.194/0.113	0.215/0.123	0.194/0.113	0.194/0.124

The best results are underlined.

**Table 5:** Performance comparison (ND/RMSE) for forecasting tasks with continuous missing.

Data	Missing	RTNMFFM	RTNMFFM(0)	TRMF(1)	TRMF	ARNMF	BTMF
(G)	60%CM	0.119/0.115	0.121/0.119	0.122/0.116	0.129/0.125	0.126/0.126	0.150/0.142
	50%CM	0.116/0.112	0.119/0.113	0.116/0.113	0.122/0.119	0.122/0.122	0.153/0.141
	40%CM	0.126/0.109	0.113/0.106	0.115/0.116	0.126/0.124	0.121/0.119	0.172/0.146
	20%CM	0.115/0.106	0.119/0.114	0.108/0.108	0.124/0.121	0.116/0.115	0.147/0.135
(H)	60%CM	0.187/0.064	0.193/0.066	0.192/0.061	0.226/0.074	0.214/0.072	0.201/0.064
	50%CM	0.207/0.071	0.198/0.066	0.191/0.060	0.202/0.063	0.204/0.068	0.180/0.058
	40%CM	0.185/0.063	0.190/0.064	0.178/0.057	0.211/0.069	0.190/0.062	0.171/0.056
	20%CM	0.172/0.058	0.178/0.059	0.188/0.058	0.186/0.061	0.186/0.062	0.176/0.058
(P)	60%CM	0.053/0.072	0.078/0.086	0.090/0.093	0.107/0.164	0.106/0.164	0.181/0.238
	50%CM	0.046/0.054	0.056/0.060	0.081/0.090	0.106/0.159	0.107/0.167	0.197/0.248
	40%CM	0.047/0.052	0.047/0.052	0.072/0.076	0.100/0.142	0.107/0.165	0.207/0.248
	20%CM	0.072/0.076	0.066/0.069	0.082/0.087	0.099/0.152	0.102/0.164	0.211/0.254
(C)	60%CM	0.243/0.069	0.284/0.076	0.290/0.076	0.321/0.080	0.305/0.081	0.475/0.112
	50%CM	0.236/0.067	0.295/0.079	0.297/0.076	0.314/0.079	0.297/0.076	0.730/0.161
	40%CM	0.243/0.067	0.282/0.074	0.290/0.075	0.312/0.078	0.270/0.073	0.401/0.097
	20%CM	0.236/0.067	0.259/0.072	0.305/0.076	0.295/0.077	0.302/0.076	0.407/0.102
(E)	60%CM	0.202/0.129	0.230/0.137	0.222/0.132	0.252/0.161	0.223/0.137	0.217/0.135
	50%CM	0.204/0.130	0.221/0.135	0.212/0.118	0.251/0.161	0.230/0.137	0.215/0.135
	40%CM	0.195/0.126	0.222/0.134	0.211/0.130	0.249/0.167	0.223/0.133	0.214/0.134
	20%CM	0.191/0.124	0.215/0.131	0.229/0.133	0.241/0.156	0.227/0.134	0.214/0.134

The best results are underlined.

## Data Availability

All data generated or analyzed during this study are included in this published article. The datasets generated and/or analyzed are available from the corresponding author upon reasonable request.

## Appendix A. Computation Time Cost

The experiments were conducted using a Windows 11 computer with Intel i7-10700 CPU and 16G RAM. The computational time cost for model training and testing on each dataset is shown in the table below.

As can be seen from Table A1, although the proposed RTNMFFM model requires more static training time, the difference is not significant.

**Table A1:** Comparison of training times for each model.

	Data (P)	Data (H)	Data (E)	Data (C)	Data (G)
RTNMFFM	10 min	33 min	3 h 28 min	2 h 26 min	1 h 42 min
RTNMFFM(0)	6 min	33 min	3 h 16 min	2 h 21 min	1 h 38 min
TRMF	5 min	32 min	3 h 00 min	2 h 12 min	1 h 36 min
ARNMF	5 min	31 min	2 h 58 min	2 h 13 min	1 h 36 min
BTMF	12 min	25 min	2 h 36 min	40 min	24 min
LSTM-ReMF	18 min	46 min	4 h 13 min	3 h 12 min	2 h 01 min

## Appendix B. The Diebold–Mariano Test for RTNMFFM and the Best Alternative Model

The Diebold–Mariano test (DM test) is used to test whether there is a significant difference in the predictive power of the two forecasting models. The Diebold–Mariano test is essentially a *t*-test that tests whether the means of the two series that produce the forecasts are equal. That is, it is a *t*-test for the zero mean of a series of loss differences. We mainly compare the results of the DM test for the two models RTNMFFM, and the best alternative algorithm at 60% RM and 60% CM. The original hypothesis of the DM test is that the two models have the same prediction results. The original hypothesis is rejected when the *p*-value of the DM test statistic is <0.05, implying that the two models have different effects, at which point the DM test statistic is <0, indicating that the RTNMFFM model is superior to the best alternative algorithm. Since the DM test is only for one-dimensional data, on each dataset, we randomly selected five of the sequences for the DM test, and the results are shown in Table A2 and Table A3. The results show that not all entries in the various datasets pass the DM test, and in most cases when the *p*-value is <0.05, the DM statistic value is indicative of a better prediction by RTNMFFM. Especially in Data (E), the ND/RMSE metrics of RTNMFFM have a clear advantage over the best alternative algorithm, while the DM test shows the same result on the five randomly selected sequences.

On the other hand, we also did the DM test on the prediction results of RTNMFFM for 60% RM and 50% RM cases, and the results are shown in Table A4. The results show no significant difference between the DM tests for 60% and 50% random missing RTNMFFM predictions. We would like to state here that the DM test is a statistical test to determine which of the univariate models has better forecasting results. Due to the lack of reference support, we cannot use the DM test to determine which multidimensional time series forecasting model is more effective, but these results can inform future research.

**Table A2:** The DM test results for RTNMFFM and the best alternative algorithm in 60% RM.

	Data (P)		Data (H)		Data (E)		Data (C)		Data (G)	
	**DM Value**	***p*** **-Value**	**DM Value**	***p*** **-Value**	**DM Value**	***p*** **-Value**	**DM Value**	***p*** **-Value**	**DM Value**	***p*** **-Value**
No. 1	−1.57	0.176	−2.89	0.004	−1.65	0.098	1.18	0.237	−0.67	0.503
No. 2	−1.42	0.002	−2.83	0.004	−3.76	0.000	−1.84	0.067	−2.62	0.010
No. 3	−2.47	0.049	1.18	0.234	−2.06	0.039	−2.31	0.022	−5.01	0.000
No. 4	−2.46	0.057	0.74	0.454	−3.97	0.000	−2.14	0.033	0.63	0.530
No. 5	−0.92	0.398	2.03	0.042	−1.90	0.057	−2.65	0.008	1.05	0.293

**Table A3:** The DM test results for RTNMFFM and the best alternative algorithm in 60% CM.

	Data (P)		Data (H)		Data (E)		Data (C)		Data (G)	
	**DM Value**	***p*** **-Value**	**DM Value**	***p*** **-Value**	**DM Value**	***p*** **-Value**	**DM Value**	***p*** **-Value**	**DM Value**	***p*** **-Value**
No. 1	0.48	0.649	−4.98	0.000	−5.57	0.000	−2.87	0.005	−1.79	0.077
No. 2	−0.85	0.431	1.37	0.170	−11.21	0.000	−3.81	0.000	−1.83	0.069
No. 3	2.51	0.054	7.21	0.000	−10.19	0.000	−0.49	0.623	−4.05	0.001
No. 4	2.09	0.090	−0.19	0.840	−2.26	0.000	−1.19	0.232	2.48	0.014
No. 5	−0.199	0.367	0.96	0.333	−4.21	0.032	−2.71	0.007	−2.15	0.033

**Table A4:** The DM test for 60% and 50% random missing RTNMFFM predictions.

	Data (P)		Data (H)		Data (E)		Data (C)		Data (G)	
	**DM Value**	***p*** **-Value**	**DM Value**	***p*** **-Value**	**DM Value**	***p*** **-Value**	**DM Value**	***p*** **-Value**	**DM Value**	***p*** **-Value**
No. 1	1.14	0.304	1.37	0.169	−2.95	0.001	7.29	0.001	−0.14	0.889
No. 2	0.25	0.819	−5.08	0.000	−4.86	0.000	6.65	0.002	0.99	0.923
No. 3	−1.29	0.253	−1.29	0.253	−1.35	0.175	4.65	0.006	6.44	0.000
No. 4	1.11	0.317	1.11	0.317	−6.47	0.001	4.44	0.000	−1.21	0.226
No. 5	−0.17	0.871	−0.16	0.872	−1.65	0.100	11.12	0.000	−2.43	0.016
